# Determining the total cost of ownership and end user perception of the Kenya National Cancer Registry (NaCaRE-KE): a DHIS2- based digital health System

**DOI:** 10.1093/oodh/oqaf007

**Published:** 2025-03-28

**Authors:** Nelly Nyaga, Elias Melli, Martin Mwangi, Milka Gicheso, Peder Digre, Steven Wanyee

**Affiliations:** Health Informatics Research and Innovation Department, IntelliSOFT Consulting Limited, George-Padmore Road, 40664-00100, Nairobi, Kenya; CEO, National Cancer Institute of Kenya, Ragathi Road, Nairobi, 30016-00100, G.P.O, Nairobi, Kenya; Consultant, Health Informatics Research and Innovation Department, IntelliSOFT Consulting Limited, George-Padmore Road, 40664-00100, Nairobi, Kenya; Instructional Design and Training Consultant, IntelliSOFT Consulting Limited, George-Padmore Road, Nairobi 40664-00100, Nairobi, Kenya; Product and Market Advancement, PATH, 437 N 34th Street, Seattle, WA 98103, USA; Health Informatics Research and Innovation Department, IntelliSOFT Consulting Limited, George-Padmore Road, 40664-00100, Nairobi, Kenya

**Keywords:** digital health, cancer registry, total cost of ownership, Kenya, digital transformation, DHIS2

## Abstract

The digital transformation of healthcare systems holds immense potential for improving healthcare delivery and achieving better health outcomes, particularly in low- and middle-income countries faced with numerous healthcare system challenges. The National Cancer Registry of Kenya (NaCaRe-KE) system is aimed at streamlining cancer surveillance data collection to inform scientific research and cancer control interventions in Kenya. This study aimed to estimate the total cost of ownership (TCO) of the NaCaRe-KE system across five facilities with varied characteristics in Nairobi County, Kenya and the National Cancer Institute of Kenya, providing insights into the financial requirements of developing and maintaining a comprehensive digital cancer registry. Leveraging quantitative methods using the Digital Square/PATH TCO tool and key informant interviews, the data revealed significant variations in the TCO based on facility size, service scope and ownership. Qualitative analysis of stakeholder interviews highlighted the perceived effectiveness of NaCaRe-KE in improving operational efficiency, although challenges such as staffing shortages and technical issues limiting its effective implementation were noted. Recommendations for enhancing NaCaRe-KE’s utility and sustainability include addressing technical challenges, enhancing user training and promoting institutional investment in IT support. Overall, this study contributes to our understanding of the financial dynamics and operational implications of digital cancer registries and provides evidence-based investments in digital health interventions in Kenya and beyond.

## INTRODUCTION

The digital transformation of health systems is now increasingly recognized as a powerful approach to address the challenges faced by health systems globally, particularly in low- and middle-income countries, to improve healthcare delivery and achieve better health outcomes [1]. The Africa Centers for Disease Control and Prevention (Africa CDC) has acknowledged the potential of digital interventions, services and applications in health (DISAH), in their digital transformation strategy [2]. The World Health Organization (WHO) has also emphasized the importance of digital health strategies in achieving universal health coverage and achieving the Sustainable Development Goals [3]. Costing the development, deployment and operation of these DISAH helps outline the required funding and ensures that resources from various stakeholders are directed toward the country’s health priorities.

Many countries in Africa, including Kenya, have acknowledged the significance of digital health transformation and have developed e-health policies and strategies to guide their efforts. As of 2020, at least 42 countries had a digital health strategy aimed at to harnessing the potential of information and communication technologies to strengthen health systems, improve service delivery and enhance health outcomes [4].

According to the WHO Classification of DISAH [5], registries provide a central authority for maintaining specific master data sets and are enforced by governance mechanisms [5]. Specifically, cancer registries are central to cancer surveillance, providing information on disease burden, mortality rates and survivorship data, which informs cancer research, prevention and control interventions at the population or regional level. As the morbidity and mortality rates of cancer increase along with the rising double burden of communicable and non-communicable diseases in developing countries, cancer registries have become indispensable tools in the global efforts against cancer [6].

Globally, there is a growing recognition of the importance of robust cancer registries for understanding the burden of cancer and developing evidence-based policies [7]. In sub-Saharan Africa, including Kenya, the availability of accurate and comprehensive cancer data is limited, posing challenges to effective resource allocation and policy development [8]. However, efforts are being made to improve this situation, with various African countries, including Kenya, working toward digitizing their health sectors [9].

The National Cancer Registry of Kenya (NaCaRe-KE) system, established through an Act of Parliament, represents a notable intervention in digitizing the health sector in Kenya. The NaCaRe-KE system plays a pivotal role in streamlining data collection for cancer cases, enabling the generation of accurate and comprehensive data for analysis. This falls within the purview of the National Cancer Institute (NCI) of Kenya, which is mandated to collect, analyze and disseminate all relevant cancer-related data in the country [7].

The NaCaRe-KE system was launched in 2021 to facilitate data collection and cancer diagnosis reporting in the country. This is a data reporting tool in which a subset of data from the patient file is entered following abstraction. Data collected in the system comprises patient data, including their demographics, specific cancer diagnosis, staging, mode of diagnosis, patient status at the time of diagnosis, treatment and survivorship. Despite being in its initial use stage, the NaCaRe-KE system has already shown promising results in enhancing the visibility of cancer burden and its distribution across different demographic groups, such as age, sex, ethnicity and county of origin which is anticipated to inform cancer management policies and resource allocation even as the system continues to be used more widely across the country.

This study aimed to estimate the total cost of ownership (TCO) of the NaCaRe-KE system, providing valuable insights into the financial requirements of developing, deploying and operating a comprehensive digital cancer registry. The study targeted sample facilities implementing the NaCaRe-KE system and included the costs incurred by NCI for the development, deployment and operation of the system in all cancer centers in Kenya. The findings will inform planning and resource mobilization efforts and facilitate evidence-based investments in the digital transformation of health systems in Kenya and across Africa [10].

By generating robust evidence on the cost implications of digital health interventions, this study contributes to the literature on best practices in the field and their potential impact on policy formulation. It aligns with the WHO’s global digital health strategy, which emphasizes the importance of evidence generation and documentation for promoting effective use of digital technologies in healthcare [11]. In addition, through the qualitative study targeting end users, the study evaluates the impact and benefits of the investments made on the NaCaRe-KE system to date, highlighting gaps in the existing system that would benefit from future investments.

This study aims to support informed decision making, influence policies and foster investments in digital health interventions in Kenya and other resource-constrained settings. By addressing the need to quantify the costs involved in developing, deploying and operating a comprehensive cancer registry, this study aligns with e-health policies and efforts in Kenya and contributes to the broader digital transformation of the health sector. Furthermore, it contributes to the Africa CDC’s Digital Transformation Strategy by providing evidence to fuel investments in digital health across the continent, ultimately improving health outcomes and strengthening health systems [2].

The District Health Information System 2 (DHIS2)-powered NaCaRe system was selected for this study primarily due to its flexibility, scalability and widespread use in public health data management across many low- and middle-income countries, including Kenya. DHIS2 is one of the most mature global goods for health with a strong track record of supporting health information systems, making it a cost-effective solution that aligns with Kenya’s broader digital health strategies. Its ability to integrate with other health systems, support multiple data formats and handle large datasets was particularly beneficial for a national cancer registry that requires continuous data collection and analysis [12].

## STUDY APPROACH

The study was conducted in two distinct components. The first component was quantitative, using Digital Square’s TCO tool [13] to analyze the total cost of ownership of the NaCaRe-KE system. Data were collected from various facility deployment sites, including the national instance, to elucidate the financial considerations associated with system implementation and maintenance.

The second component was a qualitative study conducted by administering a thematic questionnaire to end users. This qualitative inquiry assessed the effectiveness and impact of the NaCaRe-KE system, identified existing gaps and gathered suggestions for future advancements. By exploring end users’ perspectives, this qualitative approach aimed to provide insights into the system’s performance, highlight areas for improvement and potential avenues for further funding to enhance its utility and usability.

## MATERIALS AND METHODS

### Study sites

The study was conducted at five health facilities in Nairobi County, Kenya. These facilities were selected to represent a diversity of facilities involved in cancer care and reporting in Kenya. These included two national referral hospitals (Facilities A and D), two private hospitals (Facilities B and C) and one outpatient cancer treatment center (Facility E). Table 1 provides additional details about each facility.

**Table 1:** Description of the cancer centers where the data were collected

**Facility**	**Description**
A (Public inpatient)	• National teaching and referral hospital • Bed capacity of >500 • Specialized cancer services • High patient flow • High-staffing needs including multiple full-time health records information officers (HRIOs)
B (Private inpatient)	• A private cancer treatment center • Bed capacity = 50 • Offers both inpatient and outpatient cancer treatment • Has less patient flow than A • Lower staffing needs, including part-time HRIOs
C (Private outpatient)	• A private comprehensive cancer treatment facility • Bed capacity = 0 • Offers outpatient cancer treatment • Lower patient flow compared with A • Has one HRIO that serves all data needs at the facility
D (Private inpatient)	• A national diagnostic, treatment and referral center • Bed capacity >350 • Offer highly specialized cancer care. • High patient flow, comparable to that of A • Has three HRIOs
E (Private outpatient)	• Outpatient cancer treatment center • Bed capacity = 0 • Lower patient flow compared with A and D • Has one HRIO serving all data needs at the facility, thereby lowering operational costs
F	• National Cancer Institute • The official custodian of the NaCaRe system • Responsible for system development, management and operational costs of national projects

### Data collection

#### Quantitative data

For quantitative data on ownership costs, we leveraged Digital Square’s TCO tool, an interactive budgeting and benchmarking resource that facilitates the development of realistic budgets for digital health projects.

The TCO tool, currently available in an interactive Excel format [13], can be applied to a variety of digital health interventions to understand the typical cost breakdown and categories across different stages of implementation at a country level. Specifically, it aids in identifying expenses linked to digital health interventions targeting a broad base of end users, such as community health workers, as well as those tailored for national-level health system management. Users of the interactive Excel tool are prompted to estimate and input costs over a 5-year period related to various aspects of the digital health intervention, although the TCO offers some guidance on cost estimation. co-created with a group of digital health costing experts with funding from the Bill & Melinda Gates Foundation.

The TCO is divided into three main cost components aligned with the implementation phases: development, deployment and operations. These components align with the overall lifecycle of a digital health initiative, starting with initial planning and development, progressing through deployment and expansion and culminating in ongoing operational activities.

The TCO tool is designed to collect annual costs for the different things, e.g. development costs, operational costs etc. For this study, some facilities had the costs available as monthly costs and therefore the annual cost was computed. For some costs such as consultant costs, the monthly salary cost was used instead as this is what was available. Adaptations to the TCO are key to ensure the tool remains useful and relevant for every setting.

TCO data were collected through semi-structured, in-depth interviews with the finance leads or registry administrators at each facility. The interviews were conducted to obtain data on the costs of development, deployment and operation of NaCaRe through different cost line items outlined in Table 2. To ensure data comparability, the same team of research assistants conducted data collection at all facilities using a standardized interview guide to guide facility teams through the TCO tool. All costs were collected in the Kenyan shilling.

**Table 2:** Cost categories for each phase and the data categories provided at each facility

**Phase**	**Cost categories**	**A (Public inpatient)**	**B (Private inpatient)**	**C (Private outpatient)**	**D (Private inpatient)**	**E (Private outpatient)**	**F**
Development	Development costs						X
	Project management						X
	Needs assessment and requirements specifications						X
	Software development						X
Deployment	Equipment	X	X	X	X	X	X
	Infrastructure (solar, backup generators)		X	X			
	Implementation services	X	X	X	X	X	
	Integration and interoperability						X
	Software development and adaptation						X
	Testing						X
	New deployment training						X
Operation	Equipment replacement			X			
	Infrastructure replacement			X			
	Software licensing and subscriptions		X	X	X	X	
	Data and voice services			X		X	
	Refresher training						X
	Helpdesk support						
	Maintenance						X
	Testing						
	Transfer of ownership						
	Project management	X					x
	Transport and communication			X			
	Governance						X
	Monitoring and evaluation						X
	Procurement						

The tool was adopted in its entirety because it is fully illustrative and contains comprehensive user guides. Typically, this tool is used to estimate the TCO for an aggregate across multiple health systems [13]. However, we allowed flexibility in the data provided to accommodate the different contexts and operational modalities of the target sites. In collecting the financial data, access to the financial records was not requested, but authorized personnel provided the costs.

#### Qualitative data

Qualitative data were collected through interviews with selected persons including facility managers, data managers and health record information officers using an interview guide based on the Reach, Effectiveness, Adoption, Implementation, and Maintenance (RE-AIM) framework with questions assessing various components of the operations of the NaCaRe system, including workflow efficiencies in cancer reporting, perceived effectiveness, usability, sustainability plans and areas for improvement. The RE-AIM framework is a comprehensive evaluation tool used in public health and healthcare research to assess the impact and effectiveness of interventions. It comprises five key dimensions: Reach, Effectiveness, Adoption, Implementation and Maintenance, which are all assessed. Together, these dimensions provide a structured approach to evaluating interventions, ensuring both internal and external validity and facilitating the translation of research findings into practical, sustainable solutions for improving population health outcomes [14]. These interviews were administered to representatives from the facilities involved with the daily running or management of the cancer registry. Notably, while the qualitative and quantitative studies were conducted in parallel, in some facilities, the two sets of data were provided by different individuals, all of whom had experience working with the registry with each interview taking between 1 and 2 h.

Two research assistants were involved in coding, analyzing and interpreting the qualitative data.

### Data analysis

Inputs into the TCO are processed using built-in formulas, with the results presented in the cost summary, which provides a condensed overview of the implementation costs spanning a select period (year) organized per project phase, with breakdowns of the cost categories and their corresponding cost line items, as illustrated in Table 2.

Notably, to ensure the applicability of the tool to the NaCaRe context, we made some adjustments to the approach to determine various cost line items. For example, we estimated labor costs at a monthly rate rather than a daily rate. In addition, the cost of the internet was estimated as a percentage of rental cost in scenarios where the two components are included in a package. Lastly, we investigated costs over a 2-year rather than a 5-year period given the project timeline.

The results obtained from the qualitative interview were subjected to thematic analysis, where meaningful insights were drawn from recurring patterns, themes and trends in the responses and used to draw conclusions regarding the implementation of the NaCaRe system. The various themes identified before data collection were used to design the qualitative data collection tool (see Supplementary Material 1).

## RESULTS

### Quantitative results

Data were collected from five facilities that were diverse, ranging from two national referral hospitals with a bed capacity of over 350 to outpatient cancer care facilities, as outlined in Table 1. Based on the costs, the facilities can be grouped into high-, medium- and low-cost facilities, with an average TCO of KSh 2.8 million, KSh 1.2 million and KSh 0.6 million for the high-, medium- and low-cost facilities, respectively. Noteworthy, however, is that this may not be a perfect depiction because these costs are driven by the volume of data generated from each facility, which is a function of its size and the services offered there, including cancer diagnostics, treatment, monitoring and comprehensive care. Cost drivers for facilities are more prevalent in the deployment and operation phases, with all facilities reporting costs when acquiring equipment to support the data reporting and implementation services. In the operation phase, a key cost driver was the software licensing fees needed to maintain the equipment’s operating system and security, which was also reported across all facility types. Noteworthy is that the NaCaRe system is built on open-source technology; therefore, facilities do not incur licensing fees. As the custodians of the NaCaRe system, the NCI typically incurred the bulk of the system development, project management and training costs for targeted end users, along with continuous M&E. The cost of ownership per patient was calculated as a rough estimate using the bed capacity, e.g. 500 for Facility A and 50 for Facility B. For the outpatient centers with, the bed capacity for the facilities in the same class were used. This estimation is very rough as exact numbers of the cancer patient served per facility was not provided.

As shown in Table 3, costs were higher during the first year of implementation than during the second year, which was largely driven by deployment costs. Facilities incurred costs primarily during system deployment and day-to-day operations, while the NCI incurred development costs.

**Table 3:** Deployment, operational costs and total cost of ownership of the NaCaRe system across five cancer centers and the national cancer institute in 2021 and 2022 estimated in Kenya shillings

**Facility**	**Development cost**		**Deployment cost**		**Operational cost**		**Total cost**		**Cost/patient**	
	**2021**	**2022**	**2021**	**2022**	**2021**	**2022**	**2021**	**2022**	**2021**	**2022**
A (Public inpatient, 500 patients)	0	0	1 470 000.00	1 320 000.00	0	0	1 470 000.00	1 320 000.00	2940	2640
B (Private inpatient, 50 patients)	0	0	2 186 000.00	1 170 000.00	201 200.00	402 400.00	2 387 200.00	1 572 400.00	47 744	31 448
C (Private outpatient, 50 patients)	0	0	504 000.00	404 000.00	81 700.00	53 700.00	585 700.00	457 700.00	11 714	9154
D (Private inpatient, 350 patients)	0	0	2 980 000.00	2 700 000.00	150 000.00	150 000.00	3 130 000.00	2 850 000.00	8942.857143	8142.857143
E (Private outpatient, 50 patients)	0	0	931 000.00	906 000.00	1067.00	2133.00	932 067.00	908 133.00	18,641.34	18,162.66
**F (NCI)**	3 904 580.00	0	5 309 978.00	7 520 947.00	5 228 400.00	5 666 800.00	14 442 958.00	13 187 747.00		
**Totals per year**	3 904 580.00	0.00	13 380 978.00	14 020 947.00	5 662 367.00	6 275 033.00	22 947 925.00	20 295 980.00		
**Totals/phase**	**Development**	3 904 580.00	**Deployment**	27 401 925.00	**Operational**	6 275 042.00	**Total Cost**	43 243 905.00		

The highest costs by facilities were incurred during the deployment phase, with equipment and implementation services having the highest line-item costs. It is noteworthy that the line-item costs varied across the facilities, with some facilities including certain line items while others did not. This variation was attributed to the absence of those costs for some facilities and a lack of data in others, as illustrated in Table 4.

**Table 4:** Cost categories for each phase in the Kenya shillings

**Phase**	**Cost categories**	**A (Public inpatient)**	**B (Private inpatient)**	**C (Private outpatient)**	**D (Private inpatient)**	**E (Private outpatient)**	**F (NCI)**
Development	Project management						2 787 500
	Needs assessment and requirements Specifications						324 000
	Software development						793 080
Deployment	Equipment	25 000	1 016 000	110 000	387 000	80 000	3 579 956
	Infrastructure			30 000	280 000		
	Implementation services	150 000	2 340 000	768 000	1 170 000	5 600 000	
	Integration and interoperability						
	Software development and adaptation	2 640 000					419 880
	Testing						699 420
	New deployment training						8 131 669
Operation	Equipment replacement			45 500			
	Infrastructure replacement			21 000			
	Software licensing and subscriptions		60 000	135 000	3200	300 000	
	Data and voice services		543 600	45 000			
	Refresher training						5 390 000
	Helpdesk support						
	Maintenance						
	Testing						
	Transfer of ownership						
	Project management	25 000					577 200
	Transport and communication			50 000			
	Governance						2 464 000
	Monitoring and evaluation						2 464 000
	Procurement						

Notably, facilities reporting to the National Cancer Registry only incur costs during the system’s deployment at their facilities and during the day-to-day operations involved in the use and reporting to the registry. As shown in Table 3, the highest TCO was KSh 3.13 million, recorded in 2021 in a private inpatient specialized hospital. This was followed by public referral hospitals, where the total cost was KSh 2.387 million. Smaller health facilities, particularly those with lower bed capacities and offering only outpatient services, had lower total costs of ownership. The same trend is seen when the costs are separated into deployment and operations costs. The TCO decreased in the second year of system use because most deployment costs were incurred in the first year. When considering the cost of ownership per patient (with the bed capacity of the hospital being used as an estimate of the patient number), the cost is higher for facilities serving lower numbers of patients and lower for facilities with higher numbers of patients, which could be attributed to the economies of scale as more patients contribute to the cost of system development, deployment and day-to-day operation costs.

### Nationally projected total ownership cost

To estimate the TCO of all cancer centers in Kenya, we employed standard economic methodologies, including cost estimation and scale-up [15]. In our analysis, we first identified the deployment and operational costs incurred by each cancer center over a 2-year period (Year 1 and Year 2). Development costs have already been incurred and can be leveraged to scale to additional sites. The five cancer centers were then grouped into three categories. The first category represents public referral hospitals, the second represents private small- to medium-sized facilities and the third represents large private facilities. The cost for each facility was then calculated and multiplied by the number of cancer centers in the country that reported to the NaCaRe system during the study period. Based on these categories, we had 36, 27 and 16 facilities. This is summarized in Table 5.

**Table 5:** Summary of the total cost of ownership of the NaCaRe system in Kenya derived from extrapolating the costs from 5 facilities to 79 facilities reporting to NaCaRe -KE across the country between 2021-2022

	**Number of facilities reporting to NaCaRe**	**Total development Costs**	**Total deployment cost of all facilities**	**Total operation costs for all facilities**	**Total cost of ownership of all facilities in the country**
Public facilities	**36**	**0**	100 440 000.00	0.00	100 440 000.00
Small- to medium-sized facilities	**27**	**0**	75 330 000.00	6 679 800.00	82 009 800.00
Large private facilities	**16**	**0**	90 880 000.00	4 800 000.00	95 680 000.00
NCI	**1**	3 904 580.00	12 830 925.00	10 895 200.00	27 630 705
Total country TCO					**KSh 278 129 800.00** **(USD 2 172 889)**

These calculations estimated the TCO for all cancer centers in Kenya based on the costs observed in the sampled facilities over a 2-year period. This approach provides a comprehensive understanding of the economic implications of the implementation and maintenance of the NaCaRe system across the country.

The figures presented in Table 5 are estimates derived from data from a sample of cancer centers in Kenya. While efforts have been made to project the TCO accurately for all cancer centers in the country, it is important to acknowledge that these figures are estimates and may be subject to variation based on factors such as differences in facility size, infrastructure and operational dynamics. For instance, some costs like the equipment replacement were not captured due to the short project duration. It is also important to note that there were some key missing data particularly on the operational costs of systems in public health facilities. Therefore, caution should be exercised when interpreting these estimates and further research or validation may be necessary to obtain precise figures.

#### Qualitative interview results

Participants who included hospital administrators, health record information officers from the facilities, and NCI were interviewed to support the comprehensive assessment of the NaCaRe-KE system through stakeholder insights. The interview explored multiple facets of NaCaRe’s implementation, including its effectiveness, challenges, outcomes, user experiences, sustainability plans and valuable lessons learned for long-term maintenance. By delving into these dimensions, we provided a thorough evaluation of the impact of this digital health intervention and identified potential areas for improvement.

Qualitative data provided insights into the adoption of the NaCaRe-KE system, with stakeholders reporting positive perceptions of its usability and effectiveness in improving workflow efficiency. However, challenges such as increased workload and technical infrastructure issues were noted, impacting the overall user experience. These findings align with the implementation component, emphasizing areas for improvement in technical support and system optimization.

The maintenance dimension was addressed through stakeholder suggestions, including the need for regular data reviews, mentorship programs and sustained institutional investment in IT support. These strategies were proposed to ensure the long-term sustainability of the NaCaRe-KE system. The results are presented in Table 6.

**Table 6:** Summary of the qualitative interview findings presented in themes and subthemes

**Theme**	**Subtheme**	**Key findings**
**Introduction and general perceptions**	Perceived effectiveness of NaCaRe	Stakeholders perceive NaCaRe as effective for resource efficiency, accuracy and reporting. Concerns regarding increased workload and system costs were noted
	Workflow improvement and record-keeping	The NaCaRe system was praised for improving workflow and record-keeping through a centralized data management tool, a comprehensive data entry tool and enhanced quality assurance checks
**Challenges in using the NaCaRe system**	HR challenges	Challenges related to staffing, training and workload, impacting deployment phase and efficiency
	Technical infrastructure challenges	Technical challenges such as system downtime, lack of technical support responsiveness and the absence of autosave features were noted
	Governance and buy-in challenges	Backlog, integration issues, lack of buy-in and support highlighted as governance challenges that impact efficiency and workload
**Outcomes and benefits**	Positive outcomes and benefits	The NaCaRe system facilitates informed planning and decision-making and enhances the population-level impact of research and epidemiology
	Workflow and workload implications	Enhanced workflow noted, but integration increases workload due to mandatory reporting requirements
**User Experience**	Positive aspects of user experience	The system was found easy to use with a simple design and layout, enhancing the overall user experience
	Technical issues and challenges	Technical challenges include downtimes, login issues and comparisons with other systems for improvement
	Suggestions for improvement	Recommendations include implementing an autosave feature, clarifying cancer grading and enhancing accuracy through various means
**Recommendations and Overall Perceptions**	Despite challenges, overall satisfaction with NaCaRe was expressed, recommending its ease of use, comprehensiveness and efficiency	
**Changes in workflow and process**	Changes in workflow and processes	Data duplication challenge highlighted, recommending unique identifiers and addressing increased workload and reduced efficiency
	Employee adaptation and support	Robust training provided by NCI eases adaptation to the new system, positively influencing decision-making processes
**Influence on decision-making processes**		Increased demand for data, supporting research endeavors and positively influencing decision-making processes positively
**Sustainability and future enhancement**		Suggestions include regular data review, dissemination, mentorship and support for sustainability planning; desired features for future versions and institutional investment in IT support

Two key findings emerged from the qualitative analysis:

1. Stakeholders saw NaCaRe as effective in improving operational efficiency compared to previous paper-based reporting, despite challenges. They noted the following benefits: reduced time and resource requirements, improved data quality and enhanced reporting speed. However, some reported increases in workload because they had to report other data in other parallel programs.
2. Stakeholders generally rated usability positively, but technical issues need to be addressed. Stakeholders found the system easy to use; however, they faced challenges such as downtime and data duplication. They recommended enhancements to further improve user experience and data accuracy.

## DISCUSSION

This study provides valuable insights into the financial and operational implications of implementing and operating the NaCaRe cancer registry system in Kenya. The TCO analysis revealed significant variations based on facility type, with larger national referral facilities incurring lower costs per patient compared with smaller private and outpatient facilities. These variations are likely driven by differences in patient volume, services offered and staffing needs. Costs were highest during the first year of implementation, consistent with previous research on digital health interventions [16].

Despite encountering challenges [17] in accessing data from all initially targeted facilities due to local organizational policies and data availability issues, the data were successfully gathered from five diverse facilities, including national referral hospitals and outpatient cancer care facilities. These facilities varied in size, scope of services offered and ownership (government vs. private), providing a comprehensive overview of the financial landscape. The TCO differed significantly among the facilities, with high-, medium- and low-cost categories influenced by the volume of data generated, facility size and the spectrum of services provided, encompassing cancer diagnostics, treatment, monitoring and comprehensive care. Notably, facilities reporting to the National Cancer Registry incurred costs primarily during system deployment and day-to-day operations related to data usage and reporting. The analysis identified a clear correlation between facility size, service scope and TCO, with larger institutions and those offering comprehensive services incurring higher development costs but lower costs per patient, as these facilities tend to serve more patients than smaller ones. Furthermore, a decrease in the TCO in the second year highlights the impact of upfront deployment expenses. Overall, these findings underscore the complex financial dynamics involved in implementing and sustaining cancer registry systems across diverse healthcare settings, emphasizing the need for tailored financial planning and resource allocation strategies.

In addition, key cost drivers identified include initial system development, training and equipment purchase costs. Future studies should evaluate how increasing digital literacy might reduce training costs for DHISs, such as the national cancer registry. Additionally, equipment costs could be significantly reduced if shared across multiple digital interventions rather than each intervention requiring standalone equipment (e.g. computers).

While the NaCaRe-KE system shows significant promise in improving cancer data reporting and surveillance, integration and interoperability with other health information systems have yet to be fully realized. As noted in Table 4, the ‘Integration and Interoperability’ cost category currently records zero costs, reflecting the early stage of system implementation. However, this does not imply that interoperability is not a priority for the NaCaRe-KE system. Ensuring that digital health interventions are integrated into the wider health data ecosystem is crucial for improving data flow, decision-making and overall health outcomes [16]. The NaCaRe-KE system is designed to evolve and future developments will include integration with other national health systems in the national health information exchange.

The cancer registry costing process provides valuable insights and methodologies that can be integrated into other digital health costing efforts. Understanding the comprehensive costs involved in implementing and maintaining such a system can inform broader digital transformation interventions and help estimate the overall cost of achieving digital health transformation across various health domains.

The interview administered to stakeholders from various facilities and the NCI provided valuable insights into implementing the Kenya National Cancer Registry (NaCaRe-KE) system. Through thematic analysis of the interview results, we comprehensively understood NaCaRe’s effectiveness, challenges, outcomes, user experiences, sustainability plans and lessons learned.

Stakeholders perceive NaCaRe as effective in improving operational efficiency in their organizations. They highlight their role in reducing input resources, such as time, human resources and paper usage, while enhancing reporting accuracy and speed. The system’s contribution to improving workflow efficiency, record keeping and data quality is also recognized. However, concerns about increased workloads and additional costs highlight the need for further optimization to balance resource efficiency with operational demands.

The RE-AIM framework was specifically selected for its ability to provide a structured and comprehensive evaluation of interventions in public health, focusing on key dimensions like Reach, Effectiveness, Adoption, Implementation and Maintenance. These dimensions align well with our goal of evaluating the NaCaRe-KE system’s impact and sustainability in a real-world healthcare context. While RE-AIM may not encompass every benefit of a digital cancer registry, it offers a robust foundation for assessing both operational and health outcome-related aspects.

The effectiveness of the system was highlighted by stakeholders who reported significant improvements in data accuracy, workflow efficiency and informed decision-making. However, these benefits were offset by challenges such as increased workloads and technical issues, which require targeted interventions to optimize resource efficiency. The results of the qualitative investigation also indicated that enhancing the system usability and providing adequate training and support were key priorities.

The rate of adoption varied across facilities, with larger institutions demonstrating greater acceptance due to their capacity to accommodate the system’s operational demands. Smaller facilities faced barriers related to staffing shortages and resource constraints, underscoring the need for tailored support during the adoption phase.

Key challenges in the implementation phase included technical infrastructure issues, such as system downtimes and data duplication, as well as governance-related challenges like lack of stakeholder buy-in. Addressing these barriers is essential for optimizing system performance and ensuring a seamless integration into existing workflows.

Maintenance strategies were highlighted as critical for the system’s sustainability. Stakeholders recommended measures such as regular data audits, enhanced IT support and continuous capacity building to ensure the NaCaRe-KE system’s long-term viability. These recommendations align with the RE-AIM framework’s emphasis on ensuring the sustainability of public health interventions.

NaCaRe has influenced decision-making processes by increasing the demand for data to support research interventions and inform policy at the facility and government levels. These measures will ensure NaCaRe’s long-term impact on cancer management in Kenya.

### Study limitations

This study was limited to Nairobi County, one of the 47 counties in Kenya. However, efforts were made to ensure diversity among the selected facilities to enhance representativeness. Challenges arose in obtaining financial data from various organizations because the data were deemed sensitive and subject to institutional policies restricting sharing. Consequently, data were collected from only five out of the eight facilities intended, thus limiting the scope of our analysis.

## CONCLUSION

In conclusion, this study provides valuable insights into the financial and operational implications of developing, deploying and operating the NaCaRe system in Kenya. By analyzing data from five diverse health facilities, we estimated the TCO for a national roll-out of the NaCaRe system. The TCO analysis revealed significant cost variations based on facility type, with larger national referral hospitals incurring lower costs per patient compared to smaller private facilities and outpatient centers. These variations are likely driven by differences in patient volume, services offered and staffing needs. The highest costs were incurred during the first year of implementation, consistent with other digital health interventions.

If implemented at a national scale, the NaCaRe system could enable Kenya to obtain better data on cancer epidemiology, morbidity and mortality. This enhanced data collection will significantly inform research and development efforts and guide policymaking and resource allocation to address cancer more effectively. In addition to deployment of the NaCaRe system, investments in better and earlier diagnostics are crucial. Improved diagnostic capabilities would allow for an earlier detection and treatment of cancer, which is often diagnosed at advanced stages in Kenya. Understanding the stages at which individuals are currently diagnosed and the barriers to early detection are vital for improving outcomes and reducing the overall burden of cancer.

Overall, although the NaCaRe system has proven effective in enhancing cancer reporting and management, addressing the identified challenges and optimizing resource use are crucial for its sustainability and long-term success. Tailored financial planning and resource allocation strategies, along with investments in diagnostics and early detection, will be essential in supporting the expansion and continuous improvement of this digital health intervention across diverse healthcare settings in Kenya.

By employing the RE-AIM framework, this study provides structured insights into the financial, operational and strategic dimensions of the NaCaRe-KE system. The findings underscore the importance of addressing challenges in Adoption and Implementation while reinforcing the system’s Effectiveness and sustainability through targeted Maintenance strategies. These results offer valuable lessons for scaling digital health interventions in resource-constrained settings, aligning with global health priorities.

While patient-level costs and benefits were outside the scope of our study, as our focus was primarily on the financial and operational implications for healthcare facilities and stakeholders involved in the system’s deployment and maintenance. In future research, we recommend a deeper exploration of patient-related outcomes, including cost–benefit analyses and the system’s direct impact on patient care, to provide a more holistic evaluation of the NaCaRe-KE system’s value.

## Supplementary Material

TCO_Study_Interview_Guide_Supplement_oqaf007

## Data Availability

The data underlying this article are available in the article and in its online supplementary material.
