# Supplementary material for: Determining the total cost of ownership and end user perception of the Kenya National Cancer Registry (NaCaRE-KE): a DHIS2- based digital health System
Source: Oxf Open Digit Health. 2025 Mar 28;3:oqaf007. doi: 10.1093/oodh/oqaf007 (PMC12011077; doi:10.1093/oodh/oqaf007)
Supplement: TCO_Study_Interview_Guide_Supplement_oqaf007 [file tco_study_interview_guide_supplement_oqaf007.docx]

**SUPPLEMENTARY INFORMATION**

**Determining the Total Cost of Ownership and End User Perception of the Kenya National Cancer Registry (NaCaRE -KE): A DHIS2-based Digital Health System.**

Nelly Nyaga^1a^*, Elias Melli^2^, Martin Mwangi^3^, Milka Gicheso^1b,^ Peder Digre^4^, Steven Wanyee^1c^

^1a*^ Health Informatics Research and Innovation, IntelliSOFT Consulting Limited, Nairobi, Kenya, 40664-00100.

Nairobi. [nnyaga@intellisoftkenya.com/nellymuruginyaga@gmail.com](mailto:nnyaga@intellisoftkenya.com/nellymuruginyaga@gmail.com)

^2^ CEO, National Cancer Institute of Kenya, Nairobi, Kenya. 30016 - 00100, G.P.O Nairobi

^3^ Independent consultant, Nairobi, Kenya. 40664-00100).

^1b^ Instructional Design and Training, IntelliSOFT Consulting Limited, Nairobi, Kenya (40664-00100

^4^ Product and Market Advancement, PATH, Seattle, USA. 2201 Westlake Avenue

^1c^ Health Informatics Research and Innovation, IntelliSOFT Consulting Limited, Nairobi, Kenya, 40664-00100.

**Qualitative Data Collection Interview Guide**

The following questions are developed to assess the implementation of the Kenya National Cancer Registry (NaCaRe - KE), a DHIS2-based Digital Health System. The questions aim to delve into various dimensions of the NaCaRe system's implementation, drawing insights from the perspectives of stakeholders like yourself. As part of this assessment, we seek to understand the various aspects of implementation including effectiveness, challenges, outcomes, user experiences, sustainability plans and valuable lessons that can inform the long-term maintenance of the NaCaRe - KE system. Your insights are invaluable in comprehensively evaluating implementation of this digital health intervention. Your candid responses will contribute to a deeper understanding of the impact and potential improvements of the NaCaRe system. Thank you for your participation and for sharing your valuable perspectives.

**1. Introduction and General Perception:**

- How do you perceive the effectiveness of the NaCaRe system in improving operations within your organization?

*Probe for:*

- Reduction in input resources (less time, less human resources, less paper, less reporting platforms).

- Increase in output/impact (ability to have all data in one place at a glance, reducing the time and effort taken to understand the incidence/prevalence/survivorship of cancer at a facility/county/country).

- Ability for NCI to have the information faster (diagnostic/survivorship).

**2. Challenges and Addressing Issues:**

**-** What challenges, if any, have been encountered during the implementation of the NaCaRe system, and how have they been addressed?

- Break this question down into phases that are time-bound (e.g., 6-month or 1-year timelines for comparison).

*Probe for challenges across various aspects such as:*

- HR - availability
- tech support ie help desk
- tech infrastructure ie down time, bugs
- governance and buy in

**3. Outcomes and Benefits**

**-** Can you describe any positive outcomes or benefits that have arisen from using the NaCaRe system, beyond monetary savings?

*Probe for:*

**-** Population level impact (research, epidemiology, survivorship).

- Monetary savings for patients/facilities (reduced time, reduced paper).

*Probe for negative outcomes:*

- downtime ie delayed notifications or interruptions in cancer detection and notification processes
- worsening workflows - hence creating more work through use of technology
- data privacy/ethics - does the technology raise any ethical concerns? Have there been any data breaches?

**4. User Experience:**

**- How would you describe the user experience of interacting with the NaCaRe system?**

- What aspects of the NaCaRe system did you find most helpful or useful?

- Good user experience

- Were there any aspects of the NaCaRe system that you found difficult to use or confusing?

- No

- How did you feel about the design and layout of the NaCaRe system?

- Did you encounter any technical issues or errors while using the NaCaRe system?

- How did the NaCaRe system compare to other similar systems you have used in the past?

- Is there anything you would change about the NaCaRe system to improve the user experience?

- Would you recommend the NaCaRe system to others? Why or why not?

**5. Workflow and Process Changes:**

- What changes, if any, have been observed in the workflow and processes since the implementation of the NaCaRe system?

*Probe for:*

- Specific changes observed.

- Impact on efficiency and effectiveness.

- Benefits or drawbacks.

- Challenges or obstacles encountered.

- Adaptation of employees to the new system and support/training provided.

**6. Influence on Decision-Making Processes:**

**-** How has the NaCaRe system influenced decision-making processes within your organization?

- Probe for various aspects such as impact on stakeholders, insights, factors considered, access to information, time taken for decisions, quality of decisions, transparency, and communication.

- In what ways has the NaCaRe system impacted how decisions are made in your organization?
- How has NaCaRe affected who is involved in decision-making processes? Are new stakeholders included or excluded compared to before?
- Do you feel the NaCaRe system provides insights that help inform better decisions? If so, how?
- How has NaCaRe changed the inputs, data, or factors considered during decision-making processes?
- Has NaCaRe increased access to relevant information for decisions? If so, how has this availability of information influenced the process?
- What effect has NaCaRe had on the time it takes to make decisions? Does it allow decisions to be made faster or slower?
- In your opinion, has NaCaRe improved the quality of decisions being made? Why or why not?
- How has NaCaRe impacted the transparency and communication of decision-making processes?
- Overall, do you think the NaCaRe system has enhanced or inhibited effective decision-making? Please explain.
- Can you provide any examples of decisions that were improved or hindered by the NaCaRe implementation?
- Do you have any other perspectives to share about how NaCaRe has influenced decision-making processes?

**7. Sustainability and Future Enhancement:**

- What suggestions do stakeholders have for enhancing the utilization and impact of the NaCaRe system?

- What features/capabilities would you like to see in future versions of the NaCaRe KE system?

- How do you plan to sustain the NaCaRe - KE implementation over time?

- Has this plan been informed by any lessons in the last two years of NaCaRe’s operationalization? If yes, which ones?
